# Microarchitecture Parameters Describe Bone Structure and Its Strength Better Than BMD

**DOI:** 10.1100/2012/502781

**Published:** 2012-05-01

**Authors:** Tomasz Topoliński, Adam Mazurkiewicz, Stanislaw Jung, Artur Cichański, Krzysztof Nowicki

**Affiliations:** ^1^Faculty of Mechanical Engineering, University of Technology and Life Sciences, Kaliskiego 7 Street, 85-789 Bydgoszcz, Poland; ^2^South Tyneside Hospital, South Shields, Tyne and Wear NE 34 OPL, UK

## Abstract

*Introduction and Hypothesis*. Some papers have shown that bone mineral density (BMD) may not be accurate in predicting fracture risk. Recently microarchitecture parameters have been reported to give information on bone characteristics. The aim of this study was to find out if the values of volume, fractal dimension, and bone mineral density are correlated with bone strength. *Methods*. Forty-two human bone samples harvested during total hip replacement surgery were cut to cylindrical samples. The geometrical mesh of layers of bone mass obtained from microCT investigation and the volumes of each layer and fractal dimension were calculated. The finite element method was applied to calculate the compression force *F* causing *ε* = 0.8% strain. *Results*. There were stronger correlations for microarchitecture parameters with strength than those for bone mineral density. The values of determination coefficient *R* ^2^ for mean volume and force were 0.88 and 0.90 for mean fractal dimension and force, while for BMD and force the value was 0.53. The samples with bigger mean bone volume of layers and bigger mean fractal dimension of layers (more complex structure) presented higher strength. *Conclusion*. The volumetric and fractal dimension parameters better describe bone structure and strength than BMD.

## 1. Introduction

Until now bone mineral density (BMD) is a standard used widely in medical practice to assess bone quality [1] and indirectly its strength. Still the result of BMD does not give information about bone structure [2] and cannot alone contribute in strength assessment [3, 4]. The importance of microarchitecture structure factors has been recently emphasized [5, 6].

These reports are mainly concerned with examinations of osteoporosis as well as biomechanical tests of bone strength in order to combine the degree of osteoporosis with bone strength [7–9]. These studies have been conducted at several different levels: on whole bones or samples cut out from them [10, 11], or even on particular osteons or trabeculae [12, 13], using both human and animal bones.

Apart from BMD and microarchitecture structure factors the fractal dimension [14] is used to estimate bone strength. This dimension was used by other authors to evaluate bone structure and BMD [15–19]. However, there were no reports on fractal dimension and volume parameters with strength and comparison with BMD.

The main aim of the work was to assess the value of selected structural parameters in description of strength of bone. In our study we focused on volume of layers and fractal dimension of layers, BMD and their correlation with bone strength. 

We also wanted to find out which parameter would be better for description of bone strength.

## 2. Material and Methods

### 2.1. Specimen

We tested trabecular bone samples. Samples were collected from 42 human femoral heads, the mean age of the patients was 73 yr (range 50–91). These specimens were obtained during hip arthroplasty. The study was approved by the Local Ethic Committee. 

**Figure 1:**
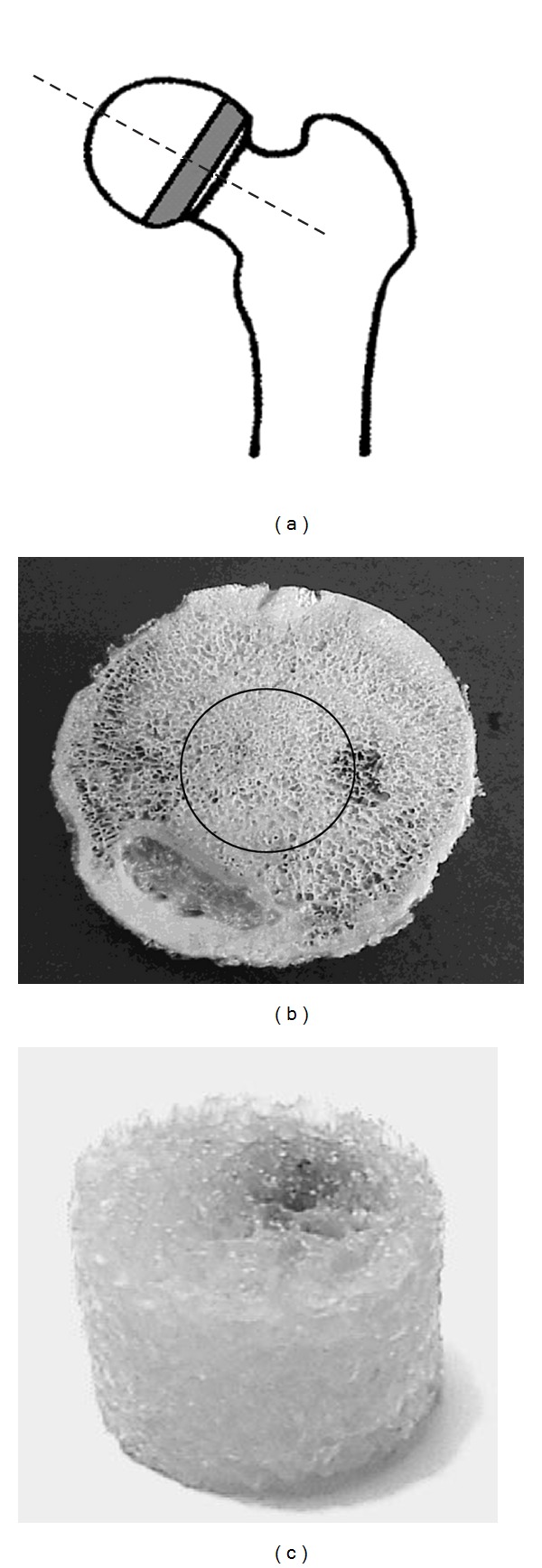
Method of obtaining sample: (a) cutting of slice; (b) cutting of sample; (c) final shape of sample.

First, slices were cut out from the base of the head at 8,5 mm thickness, perpendicular to the axis of the neck of the bone (Figure 1(a)). Then, from the central region of the slices (Figure 1(b)), the samples were cut out in the shape of a cylinder, 10 mm diameter and 8,5 mm height (Figure 1(c)).

### 2.2. MicroCT Technique

MicroCT investigation of cylindrical samples was done on microCT scanner (MicroCT 80 scanner, Scanco-Medical AG, Switzerland) with resolutions of 36 microns and with basic parameters: 70 kV, 114 *μ*A, 500 projections/180°, and 300 ms integration time. Thus we obtained around 230 scans for each sample. 

On the base of these images, the representative geometry of the sample was done using a bone reconstruction algorithm called “*hexahedron method*” [20].

In this algorithm, single layers of a model were created by comparing images of two neighboring scans. When on the same coordinate in both scans the color of pixels represented bone, voxels of bone between the scans were created. On the contrary when none or only one pixel was colored, it was omitted.

A cube was created, the so-called voxel with its base, in shape of square of side of one pixel long. Its height equals the distance between neighboring layers (cube of dimensions of 36 × 36 × 36 microns). Having checked all pairs of pixels in two specific scans the next pair of images were recorded and the whole procedure was repeated and another layer of cubes created.

### 2.3. Volume Calculation

On the basis of geometry prepared in this way, the bone volume parameters of the sample structure were calculated for every layer assigned as local volumetric parameter. It was performed by calculating number of bone voxels of known dimensions. 

For each sample volume of single layer (*V*) was calculated and, respectively, mean volume (*V*_*m*_) for the whole sample. Then, the standard deviation (SD) for *V*_*m*_ (SD_*V*_*m*__) and relative standard deviation (RSD) for *V*_*m*_ (RSD_*V*_*m*__) were calculated.

### 2.4. Fractal Dimension

To assess the complexity of the bone structure we applied fractal dimension. Fractal dimension value is between 2 and 3 for the whole sample (3D structure) and from 1 to 2 for single layer (2D structure). This results from the fact that bone mass does not fulfill completely the sample's volume but it forms a porous structure. 

Since the bone destruction occurs locally (it starts in single layer), we assumed that instead of fractal analysis for the whole sample it is better to calculate the fractal dimension for the single layers of the sample. To calculate fractal dimensions we applied box-counting method [21] using Sarkar and Chauduri's algorithm [22]. We used its extended version, that is, shifting differential box counting (SDBC) presented by Wen-Shiung et al. [23]. In this SDBC algorithm fractal dimension for box sizes (in voxels for the whole sample and in pixels—for single layers) was calculated, varying from 2 × 2 × 2 (2 × 2 for layers) up to 45% of the maximal size of microCT stack image (image size).

At each calculation stage box shifting was assumed for two voxels for the whole sample (two pixels for single layer).

Finally, the mean fractal dimension was calculated as the slope of the regression line for logarithms of box counts and sizes. The determination coefficient *R* ^2^ for the relation between the logarithms of box counts and box size was always over 0.97 for each image.

For each sample fractal dimension single layer (Df) was calculated and respectively mean fractal dimension (Df_*m*_) for the whole sample. Then, standard deviation for Df_*m*_ (SD_Df_*m*__) and relative standard deviation for Df_*m*_ (RSD_Df_*m*__) were calculated.

### 2.5. Compression Force

Based on the literature descriptive characteristics of bone material [20] and the structure of our samples (microCT of our study) we applied the finite element method (FEM). Thus we may virtually assess the force producing certain deformation of bone structure.

In our study compression force numerical analyses were performed with FEM software (Ansys 11.0 software, ANSYS Corp., Canonsburg, PA, USA). Analyses were carried out for a bone model consisting of layers, reconstructed according to the “*voxel to element”* method. The mesh characteristic for this method was prepared so a piece of a geometric structure—voxel—was directly transformed to finite element SOLID45.

**Figure 2:**
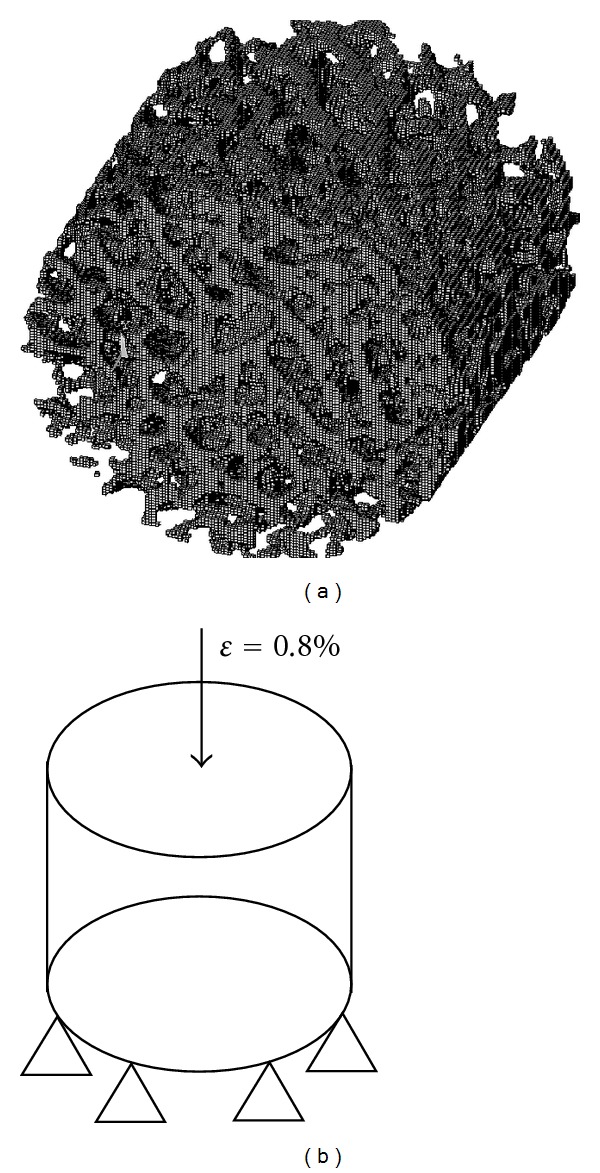
Numerical analysis of bone structure: (a) mesh; (b) schema of boundary condition.

To maintain stability of the calculation iteration process the elements not affecting the stiffness of the analyzed structure were removed from the mesh at the stage of solving the numerical problem. Also the elements that could freely turn round their axis, perpendicular to the sample cylinder axis, were removed. An example of the mesh used for numerical analysis of bone structure is presented in Figure 2(a).

For analyses of the structural character, isotropic material properties described by the tissue Young modulus *E* = 10 GPa and Poisson coefficient *ν* = 0.3 [20] were accepted. For the above assumptions the results of force calculations depend solely on the structure of the modeled tissue.

At the stage of establishing boundary conditions, the sample was virtually supported on the bottom cylinder base. At the opposite end of the cylinder, a displacement force was applied aiming to obtain the assumed strain *ε* = 0.8% (Figure 2(b)). The value of the reaction force occurring for a given displacement was the result of calculation.

### 2.6. BMD Assessment

BMD (bone mineral density) assessment was performed with dual energy X-ray absorptiometry DEXA apparatus (Lunar—Expert device (GE, WI, USA)) with projection parallel to the cylindrical sample's axis.

### 2.7. Statistics

When defining the relationships of volume of bone, fractal dimensions, and BMD with force the Pearson determination coefficients were applied. Curve fitting was performed by using Excel (Excel 2003, Microsoft, USA) software.

## 3. Results

Table 1 shows the results of BMD, Df_*m*_, and *V*_*m*_ of the samples with mean, minimal, maximal values, SD and RSD for all assessed parameters. 

The range of variability of BMD was within 0.121 to 0.404 with mean value of 0.243. This variability was within the range between 50% and 166% of the mean value of the BMD.

 The range of variability of mean fractal dimension was within 1.302 to 1.702 with mean value of 1.567. This variability was within the range between 83.1% and 109% of the mean value of the fractal dimension.

The range of variability of mean bone volume of the layers was within 0.155 to 0.944 mm^3^, with mean value 0.531 mm^3^. This variability was within the range from 29% to 178% of the mean value of the volume.

**Figure 3:**
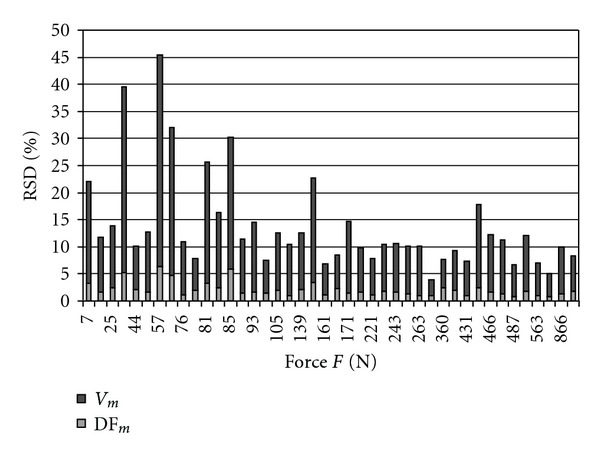
Values of RSD for Df_*m*_ and *V*_*m*_.

The values of relative standard deviation (RSD) for Df_*m*_ and *V*_*m*_ for every sample are showed in Figure 3. The sample variability of the fractal dimension of the layers of the samples described by relative standard deviation RSD_Df_*m*__ was smaller than RSD_V_*m*__. The highest values of RSD for both parameters are observed mostly for samples with relative small values of force *F*. The values of RSD show similar dynamic of its change.

**Figure 4:**
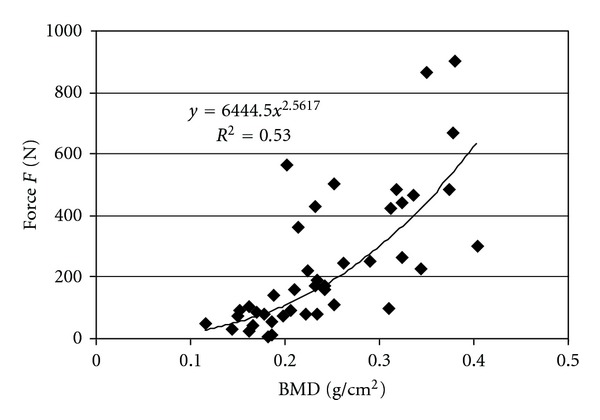
Relation between force *F* and bone mineral density BMD.

**Figure 5:**
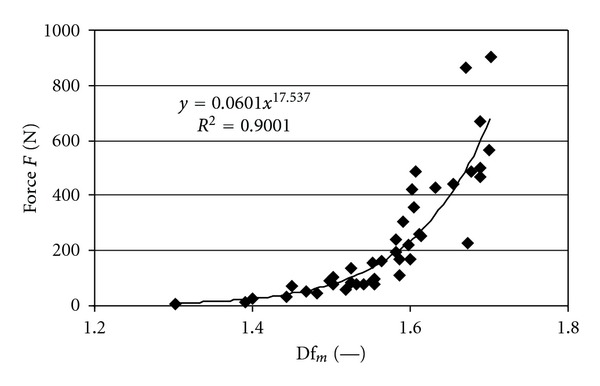
Relation between force *F* and mean fractal dimension Df_*m*_.

**Figure 6:**
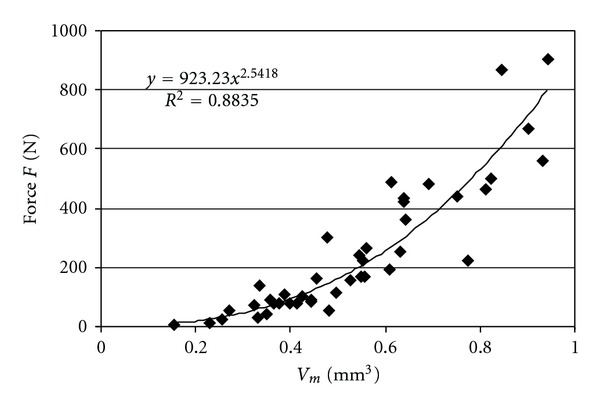
Relation between force *F* and mean volume *V*_*m*_.

In Figures 4–6 the relations between the BMD, mean fractal dimension, mean volume, and compression force *F* are presented. In Table 2, we present the values of the determination coefficients *R* ^2^ for this relation when utilizing linear regression to describe this relation with exponential function. The highest values of determination coefficient were obtained for relation between the mean fractal dimension Df_*m*_ and force *F* (*R* ^2^ = 0.9, *P* value 2.973*·*10^−10^) and the mean volume *V*_*m*_ (*R* ^2^ = 0.88, *P* value 3.338*·*10^−15^) and force, *F*. For BMD and force *R* ^2^ was 0.53 (*P* value 6.587*·*10^−8^).

To show the differences in structure of samples three specimens were taken, assigned as sample 1–3. The criterion of choice was similar BMD value (Table 3).

The volume of these samples differ significantly—mean volume of sample 3 almost doubles that of sample 1.

They also have different Df_*m*_. There are slightly different RSD_*V*_*m*__ and RSD_Df_*m*__ for sample 1 and sample 2, and there is big difference between those values and the values for sample 3. The differences in value of force *F* are visible and they are in keeping with conclusion from Figures 5–6 that with increasing Df_*m*_ and *V*_*m*_ the value force *F* also increases.

**Figure 7:**
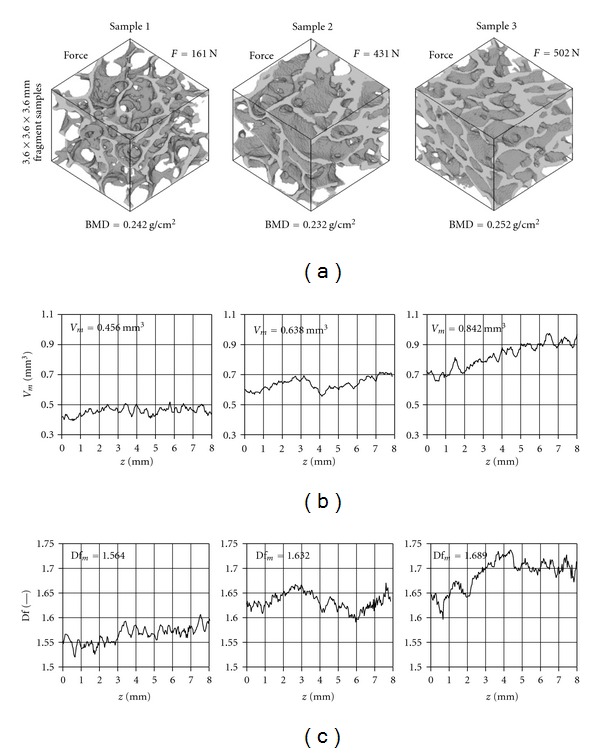
The structure of three selected samples: (a) curves of changes of volume *V*; (b) fractal dimension Df (c) along the axes of these samples.

In Figure 7(a) we present selected fragments (3.6 × 3.6 × 3.6 mm) of these three samples with different structure. The graphic presentation of volume (Figure 7(b)) and fractal dimension variability (Figure 7(c)) for every layer at sample height *z* are showed. Despite similar BMD, the structure of the samples is different. The *V* and Df curves are similar (they show almost the same dynamic of change).

## 4. Discussion

Until now BMD has been one of the major parameters used widely in medical practice to assess bone quality and indirectly the risk of fracture. Although the result of BMD gives the information on bone density, it does not give information about bone structure and its susceptibility to break. Bone mineral density shows low sensitivity and specificity, as over 50% of fractures occur in persons without osteoporosis in BMD exam and most women with osteoporosis assessed with this method do not sustain a fracture [24]. Langton et al. state that currently there is no accurate noninvasive measure of overall bone strength [25].

We assumed that bone structure is not homogenous, and certain areas are less filled up with bone mass. We think that the process of breaking is initialized in some areas with lower strength. Thus, our studies considered microstructural level of bone (defined as 36-micron layer of bone). We assessed BMD, bone quantity in layer (expressed as volume of bone in layer), and bone structure (fractal dimension). Then, we combined these parameters with compression force.

Although some papers reported on bone structure parameters and osteoporotic fractures [24, 26] and evaluation of bone layers [27], we were not able to find any paper about the correlation of bone volume in the layer with force and correlation of fractal dimension (2D) with this force and simultaneously we compare the results of BMD of the whole sample with force. Thus, we planned to assess possible utility of these structural parameters in bone strength description.

In our study we assumed that the force (*F*) caused 0.8% strain [28] which corresponded with the elastic range of strain trabecular bone. Based on force *F* value it is possible to estimate indirectly the strength of the bone. Elastic modulus gives valuable information on the possible impending fracture as strain above this value causes the damage of microstructure. Accumulation of these damages leads to clinically visible fractures.

 The variability of Df measured by RSD changes corresponds with variability of volume *V* in all samples (Figure 3). Values of RSD for Df_*m*_ and *V*_*m*_ decrease with growth of the value of force *F*. It suggests that the samples with smaller variability in structure prove greater strength. In the samples with high variability there are layers with significantly different strength and cracking begins in some areas with lower strength (samples with high RSD_Df_*m*__ and RSD_*V*_*m*__ values). Thus, more homogenous structure is more resistant to microstructure damage. 

Like Seeman [29] we also found that volume and strength are correlated better than BMD and strength. However, in his study these parameters were considered in the whole sample. Also Bousson et al. [30] found that for low BMD values local, that is, microscopic variables contribute more to bone strength than macroscopic ones. 

The relations of *V*_*m*_ and Df_*m*_ with force are similar (Figures 5–6). Thus, the samples with bigger mean bone volume of layers and bigger mean fractal dimension of layers (more complex structure) showed greater strength. On the contrary BMD displayed weaker tendency for increase in the whole range of change of force *F* (Figure 4).

When comparing variability of two microstructural parameters we see that the values of RSD_*V*_*m*__ are bigger than RSD_Df_*m*__. This might mean that relative scatter for fractal dimension is narrower, thus in diagnostic procedure fewer measurement data of fractal dimension than of volume are sufficient to conclude about bone structure. Df_*m*_ is more sensitive when compared with mean volume *V*_*m*_.

To assess which parameter BMD, volume of layers, or fractal dimension of layers, describes the strongest relation with force it is best to utilize determination coefficient *R* ^2^ (Table 2). The highest determination coefficients are for the relations of mean volume with force and mean fractal dimension with force. In our study *R* ^2^ for these relations was around 0.9. On the contrary the determination coefficient for BMD with force was clearly lower, −0.53.

Low correlation between BMD and destructive stress for shearing, compression, and tension *R* ^2^ = 0.37 was also founded by Zioupos et al. [31]. Thus, BMD is less useful for description of the force causing bone deformation than mean volume of bone layers and mean fractal dimension.

When analyzing *R* ^2^ for correlation of Df_*m*_ and *V*_*m*_ with force *F* one can conclude that strength of bone is more dependent on the complexity of the trabecular structure than on the volume of bone tissue in a volume of bone. In other words, among two samples of similar volume, greater strength characteristics should be showed by the one which presents more developed trabecular architecture.

To visualize our conclusions three samples of similar BMD are presented in Figure 7. Their mean volumes, fractal dimensions, and forces (Table 3) were clearly different and so was relative standard deviation.

If we accepted that BMD was a good descriptor of structural change [1, 32–34], one would expect similar structure in all three samples. However both data from Table 3 and images (microCT, Figure 7) show otherwise. Again the examples of these three samples support our analyses and that of another report [3] that BMD result is not the best descriptor of bone fracture risk.

Our study shows certain limitation as we assessed trabecular bone only. A similar study with cortical bone should be performed and the results be confronted with our findings. We still feel strong to publish the results based on trabecular bone only in order to share our doubts on BMD reliability in bone quality assessment.

## Figures and Tables

**Table 1:** Bone mineral density, fractal dimension and volume of the bone layer related to applied force.

Value	BMD (g/cm^2^)	Df_*m*_, —	*V*_*m*_, (mm^3^)	*F*, (N)
mean	0,243	1,567	0,531	245
min	0,121	1,302	0,155	7
max	0,404	1,702	0,944	904
SD	0,080	0,099	0,199	225
RSD, %	33	6	37	92

**Table 2:** Strength of correlation expressed as determination coefficient *R* ^2^ of force *F* relation mean volume *V*_*m*_, mean fractal dimension Df_*m*_, and BMD.

Determination coefficient *R* ^2^ for relation			
Relation	*V*_*m*_ versus *F*	Df_*m*_ versus *F*	BMD versus *F*
Value *R* ^2^	0.88	0.9	0.53

**Table 3:** Microarchitecture characteristics of selected samples with similar bone mass density.

Samples	BMD	*V*_*m*_	RSD_*V*_	Df_*m*_	RSD_Df_	*F*
	g/cm^2^	mm^3^	%	—	%	N
Sample 1	0.242	0.456	5.91	1.564	1.07	161
Sample 2	0.232	0.638	6.33	1.632	1.06	431
Sample 3	0.252	0.842	10.32	1.689	1.85	502
